# Magnitude and factors associated with nonadherence to antiepileptic drug treatment in Africa: A cross‐sectional multisite study

**DOI:** 10.1002/epi4.12052

**Published:** 2017-03-30

**Authors:** Fredrick Ibinda, Peter Odermatt, Symon M. Kariuki, Angelina Kakooza‐Mwesige, Ryan G. Wagner, Seth Owusu‐Agyei, Honorati Masanja, Anthony K. Ngugi, Caroline K. Mbuba, Victor C. K. Doku, Brian G. Neville, Josemir W. Sander, Charles R. J. C. Newton, the SEEDS writing group, Rhian Twine, Myles Connor, F Xavier Gómez Olivé, Mark Collinson, Kathleen Kahn, Stephen Tollman, Alexander Mathew, George Pariyo, Stefan Peterson, Donald Ndyomughenyi, Rachael Odhiambo, Eddie Chengo, Martin Chabi, Evasius Bauni, Gathoni Kamuyu, Victor Mung'ala Odera, James O Mageto, Ken Ae-Ngibise, Bright Akpalu, Albert Akpalu, Francis Agbokey, Patrick Adjei, Christian Bottomley, Immo Kleinschmidt, Steve White, Thomas Nutman, Patricia Wilkins, John Noh

**Affiliations:** ^1^ Centre for Geographic Medicine Research (Coast) Kenya Medical Research Institute Kilifi Kenya; ^2^ Studies of Epidemiology of Epilepsy in Demographic Surveillance Systems (SEEDS)—INDEPTH Network Accra Ghana; ^3^ Department of Public Health and Epidemiology Swiss Tropical and Public Health Institute Basel Switzerland; ^4^ University of Basel Basel Switzerland; ^5^ Iganga‐Mayuge Health and Demographic Surveillance System Kampala Uganda; ^6^ Department of Paediatrics and Child Health Makerere University College of Health Sciences Kampala Uganda; ^7^ MRC/Wits Rural Public Health & Health Transitions Research Unit (Agincourt) School of Public Health Faculty of Health Sciences University of the Witwatersrand Johannesburg South Africa; ^8^ Department of Public Health and Clinical Medicine Epidemiology and Public Health Sciences Umeå University Umeå Sweden; ^9^ Kintampo Health Research Centre Kintampo Ghana; ^10^ Ifakara Health Institute Ifakara Tanzania; ^11^ Research Support Unit Faculty of Health Sciences Aga Khan University—East Africa Nairobi Kenya; ^12^ Department of Public Health School of Medicine and Health Sciences Kenya Methodist University Meru Kenya; ^13^ Institute of Psychiatry Kings College London London United Kingdom; ^14^ Neurosciences Unit Institute of Child Health University College London London United Kingdom; ^15^ NIHR University College London Hospitals Biomedical Research Centre UCL Institute of Neurology Queen Square United Kingdom; ^16^ Epilepsy Society Chalfont St. Peter United Kingdom; ^17^ Stichting Epilepsie Instellingen Nederland (SEIN) SW Heemstede the Netherlands; ^18^ Department of Psychiatry University of Oxford Oxford United Kingdom

**Keywords:** Antiepileptic drugs, Adherence, sub‐Saharan Africa, Epilepsy, Treatment gap

## Abstract

**Objectives:**

The epilepsy treatment gap is large in low‐ and middle‐income countries, but the reasons behind nonadherence to treatment with antiepileptic drugs (AEDs) across African countries remain unclear. We investigated the extent to which AEDs are not taken and associated factors in people with active convulsive epilepsy (ACE) identified in cross‐sectional studies conducted in five African countries.

**Methods:**

We approached 2,192 people with a confirmed diagnosis of ACE for consent to give blood voluntarily. Participants were asked if they were taking AEDs, and plasma drug concentrations were measured using a fluorescence polarization immunoassay analyzer. Information about possible risk factors was collected using questionnaire‐based clinical interviews. We determined factors associated with nonadherence to AED treatment in children and adults, as measured by detectable and optimal levels, using multilevel logistic regression.

**Results:**

In 1,303 samples assayed (43.7% were children), AEDs were detected in 482, but only 287 had optimal levels. Of the 1,303 samples, 532 (40.8%) were from people who had reported they were on AEDs. The overall prevalence of nonadherence to treatment was 63.1% (95% confidence interval [CI] 60.5–65.6%) as measured by detectable AED levels and 79.1% (95% CI 73.3–84.3%) as measured by optimal AED levels; self‐reported nonadherence was 65.1% (95% CI 45.0–79.5%). Nonadherence was significantly (p < 0.001) more common among the children than among adults for optimal and detectable levels of AEDs, as was the self‐reported nonadherence. In children, lack of previous hospitalization and learning difficulties were independently associated with nonadherence to treatment. In adults, history of delivery at home, absence of burn marks, and not seeking traditional medicine were independently associated with the nonadherence to AED treatment.

**Significance:**

Only about 20% of people with epilepsy benefit fully from antiepileptic drugs in sub‐Saharan Africa, according to optimum AEDs levels. Children taking AEDs should be supervised to promote compliance.

**Key Points**

- Only about 20% of persons with epilepsy in Africa benefit fully from antiepileptic drugs (AEDs)
- Nonadherence was greater in children than in adults
- The reasons for the significantly higher rate of nonadherence to AEDs found in children compared to adults are unclear
- Status epilepticus was more common in children, which may be a consequence of poor seizure control owing to nonadherence in children

Epilepsy is ubiquitous but appears to be more prevalent in resource‐poor settings.^1^ It is associated with morbidity and premature mortality,^2^ and it exerts a considerable burden on health systems, especially in low‐income countries.^3^ People with epilepsy have poor social and health outcomes.^4^, ^5^ They may also have behavioral problems,^4^ learning difficulties, and neurological deficits and may frequently be burned.^5^ They are often stigmatized, which is associated with lower socioeconomic status, poor marriage prospects,^5^ and poor performance in those attending school.^6^

Epileptic seizures can be successfully controlled in up to 70% of people using relatively inexpensive antiepileptic drugs (AEDs).^7^ Most people with epilepsy in Africa,^8^ however, do not seek biomedical treatment. Additionally, many of those who are prescribed treatment do not adhere to it, which contributes to the epilepsy treatment gap, hereafter referred to as nonadherence to AED treatment or as nontaking of AEDs^8^, ^9^ and defined as the proportion of people with active epilepsy whose seizures are not treated appropriately expressed as a percentage.^10^ There is some evidence that premature mortality is lower and disability‐adjusted life years fewer in those taking AEDs regularly.^2^, ^11^ The extent of nonadherence and associated factors are not fully understood in Africa, probably because of lack of facilities to measure AED levels.

Two studies in Kenya measured nonadherence on the basis of either self‐reports or blood levels, with the latter determined from detectable or optimal levels of AEDs.^12^, ^13^ The findings of these studies cannot be generalized to other African settings, where causes and beliefs about epilepsy may differ.^14^ Self‐reported nonadherence is easy, inexpensive, and convenient to measure, but it is an insensitive measure with poor specificity compared to nonadherence measured by blood levels.^13^, ^15^ It is important to validate the self‐reported nonadherence against AED levels from other settings in Africa. Little is known about the factors associated with nontaking as measured by optimal levels, especially in sub‐Saharan Africa.

We assessed the magnitude of nonadherence as measured by AED levels in people with active convulsive epilepsy (ACE) from five sites in Africa. We also investigated factors associated with optimal drug levels. We hypothesized that nonadherence prevalence and associated factors will differ according to site because there may be cultural, economic, and health‐seeking behavior differences across the sites.

## Methods

### Study setting

This study was nested within large population‐based cross‐sectional studies in five Health and Demographic Surveillance Systems (HDSSs), which are part of the International Network for the Demographic Evaluation of Populations and Their Health (INDEPTH) (http://www.indepth-network.org/). These studies employed a three‐stage screening methodology to identify people with ACE in Kilifi, Kenya (lower‐middle‐income country); Agincourt, South Africa (upper‐middle‐income country); Iganga‐Mayuge, Uganda (low‐income country); Ifakara, Tanzania (low‐income country), and Kintampo in Ghana (lower‐middle‐income country) (databank.worldbank.org/data/download/site‐content/CLASS.xls).^16^, ^17^

Briefly, Kilifi HDSS is located in a rural area on the Kenyan coast, has a population of about 280,000 in an area of 891 km^2^, and hosts studies of childhood infections and neurological/mental health disorders. Agincourt HDSS is located in a semiarid area on the northeast of South Africa, covers 420 km^2^, has a population of 82,795, and has a mortality rate of 22/1,000. Iganga‐Mayuge HDSS is located near the shores of Lake Victoria, with a population of about 64,143, and hosts studies on neonatal and childhood infections. Ifakara HDSS is located in rural southern Tanzania, with a population of 93,423, and hosts studies on childhood infections and maternal/child health. Kintampo HDSS is located in central Ghana, in an area of 3,162 km^2^, has a mortality rate of 7.8/1,000, and conducts studies on mental health problems.^5^, ^17^

### Study participants and procedures

The study population consisted of people with a confirmed diagnosis of ACE who were identified from community cross‐sectional surveys conducted between August 2008 and April 2011 (1,711 people)^17^ and those who had earlier concealed their epilepsy status in the surveys but later presented to epilepsy clinics within the study sites during the study period (481 people).^5^ Phenobarbital, phenytoin, carbamazepine, and sodium valproate are the AEDs most commonly available in main public and private hospitals and pharmacies, although the supply may be erratic. A total of 2,192 people were visited in their homes by a trained fieldworker and asked to consent to participate. Those who consented were invited to their local assessment centers, where data on possible risk factors was collected through interview‐based questionnaires on sociodemographic and clinical characteristics. The parent or caregiver was interviewed if the person with epilepsy was a child or cognitively impaired. Blood samples were taken from the participants following an informed consent. ACE was defined as the presence of at least two unprovoked convulsive seizures, with at least one seizure within the previous 12 months, which are the criteria for starting AED treatment in many parts of Africa.^17^ Focal seizures were defined as those involving one part of the body; frequent seizures as those occurring daily; and status epilepticus as seizures lasting for at least 30 min.^5^, ^18^, ^19^

People were asked if they were taking AEDs (with tablets of the different AEDs shown on a board), which was defined as self‐reported adherence. The self‐reported nonadherence was calculated as the proportion of those diagnosed with epilepsy who said they were not on medication. Blood samples were assayed for the most commonly used AEDs at the five sites (phenobarbital, phenytoin, carbamazepine, and sodium valproate) on basis of the AEDs that are prescribed at each site (sodium valproate was only assayed for the South African site). At each site, blood was collected in 4‐ml heparin tubes and placed in racks sealed in plastic bags. The racks with the blood tubes were placed in secured transport boxes with cool packs and then were transported to the site laboratory within 6 h and stored in −80°C freezers. The transport boxes were then collected by a courier at an agreed upon time for transportation to Kilifi. Plasma drug concentrations were measured using a fluorescence polarization immunoassay analyzer (TDxFLx Abbott Laboratories, Abbott Park, IL, U.S.A.). For standardization, all assays were done at one site (Kilifi, Kenya). Nonadherence was calculated as the proportion of those diagnosed with epilepsy but not on appropriate biomedical treatment for seizures—either they do not seek biomedical treatment or do not adhere to the prescribed regimes—expressed as a percentage.^8^, ^9^, ^10^ The detectable ranges for the different drugs were classified as follows: phenobarbital 1.1 μg/ml, phenytoin 1.0 μg/ml, carbamazepine 0.5 μg/ml, and sodium valproate 1.0 μg/ml. The optimal ranges were: phenobarbital 10–40  μg/ml, phenytoin 10–20  μg/ml, carbamazepine 4–11  μg/ml, and sodium valproate 50–120 μg/ml.^13^, ^15^, ^20^ Nonadherence, as measured by detectable and optimal AED levels, was calculated as the proportion of those diagnosed with epilepsy who had lower than detectable levels to those who had optimal levels of AEDs. The sensitivity of self‐reported nonadherence was computed as the proportion of those without detectable AED levels who reported to the clinician that they did not take AEDs during the clinical history stage, and the specificity was computed as the proportion of those with detectable AED levels who reported taking them.

Because the magnitude of nonadherence from Kilifi has been previously published,^13^ we used AED level data from this site together with similar data from other sites to determine the overall extent of nonadherence, to compare the site‐specific nonadherence measures, and to investigate the factors associated with not taking the AEDs across all the five sites. In Kilifi only factors associated with detectable but not optimal levels of AEDs have been reported previously,^12^, ^13^ so we measured associations for optimal levels for all the five sites. We investigated factors (Table S1) associated with nonadherence across the sites to determine whether similar interventions would help improve taking of AEDs. The investigated factors in Table S1, including burns, previous hospitalization, and learning difficulties were obtained through standard questionnaire‐based interviews or clinical examinations performed by trained epilepsy clinicians. The prevalence of nonadherence in Kilifi was 62.4% for detectable levels of AEDs and 81.6% for optimal drug levels, while the self‐reported adherence was 73.7%.^13^

### Statistical analysis

The data were double entered and verified in MySQL Version 5 open‐source database (Oracle Corporation, Redwood Shores, CA, U.S.A.). All analyses were performed using R, an open‐source software for statistical computing and graphics (version 3.1.2).^21^ The Pearson chi‐square test or (Fisher's exact test where appropriate) was used to examine the distribution of participants' characteristics across the five sites. The Wilcoxon rank‐sum test was used to compare the median ages of the participants across the five sites. Nonadherence to treatment was defined as the proportion of people with epilepsy (PWE) not on appropriate AEDs based on self‐reports, detectable AED levels, or optimal AED levels. The overall prevalence of nonadherence (as measured by AEDs detectable in plasma and from self‐reports) was modeled using multilevel logistic regression because this accounts for potential clustering within the sites (the site was taken as the second level in the multilevel model), which could result in underestimation of standard errors in model coefficients. The degree of heterogeneity of adherence from PWE from the same site was computed using the intraclass correlation coefficient (ICC).^22^ To obtain the nonadherence estimate from the model, we applied the inverse of link function. The multilevel models were fitted using the *lme4 package* in R, and the *arm* package^23^ was used to compute the confidence intervals.^24^

Multilevel logistic regression (with site as the second level) was used to investigate the factors (that could be reliably recorded in all sites) associated with nonadherence based on optimal AEDs levels (Table S1). For each predictor, a univariable model was fitted initially to identify variables for a multivariable multilevel model aimed at ascertaining variables that were independently associated with nonadherence. Only variables with p values < 0.25 were included in the multivariable models. We examined whether the nonadherence was influenced by the differences in the included explanatory variable across the sites using the likelihood ratio test.^22^ Results from the models that fit the data better across the sites were reported. Variables that met criteria for inclusion into the multivariable model (p < 0.25) were further investigated. The odds ratios (ORs) and their corresponding 95% confidence intervals (95% CIs) are reported. These analyses were done separately for children (<18 years) and adults (≥18 years) because AED use is thought to be different between the two groups.^3^ A p value ≤ 0.05 is considered significant.

### Ethical approval

This study was approved by the local institutional ethical committees from the five sites and by the ethics committee at the Institute of Child Health, University College London, United Kingdom. Written informed consent was obtained for each participant.

## Results

### Characteristics of the participants

Of the 2,192 who had been diagnosed with ACE, 1,303 (59.4%) consented and provided a blood sample. There were no significant differences between those who provided blood and those who did not apart from an overrepresentation of people without neurological deficits and those with focal seizures in those who gave blood (Table S2), likely to be those with ACE perceived as less severe. There was equal representation of men and women (672 vs. 631, p = 0.52; Table S2), but many other sociodemographic and clinical factors from across the sites differed in children and adults (Tables S3 and S4).

### Antiepileptic drugs assayed

The samples were assayed for phenobarbital (1,145/1,303, 87.9%), carbamazepine (313/1,303, 24.0%), phenytoin (191/1,303, 14.7%), and sodium valproate (15/1,303, 1.2%). Overall, 532/1,303 (41.0%) people with ACE reported using AEDs: 397 of the 532 (74.6%) ever recalled using phenobarbital; 146 (27.4%), carbamazepine, 118 (22.2%), phenytoin; 12 (2.3%), diazepam; and 78 (14.7%), sodium valproate. AEDs were detected in 339/532 (63.7%), with 253/339 (74.6%) on monotherapy. Table S5 shows the distribution of detected AEDs compared to those reported. AEDs were detected in 143 (18.5%) of those who said they were not on medication.

### Sensitivity and specificity of self‐reported nonadherence

The sensitivity of the self‐reported nonadherence as measured against detectable blood levels of AEDs for all sites together was 76.5% (95% CI 73.4–79.4%) and the specificity was 70.3% (95% CI 66.0–74.4%). Sensitivity was highest in Kintampo 91.1% (85.8–94.9%) and lowest in Agincourt 58.0% (95% CI 47.7–67.8%) (Table 1). Specificity varied across the sites, being highest in Kilifi 94.7% (95% CI 90.5–97.4%) and lowest in Kintampo 18.4% (95% CI 11.3–27.5%).

**Table 1:** Site‐specific and the overall estimates of nonadherence to antiepileptic drugs

Site	Agincourt	Ifakara	Iganga‐Maguye	Kilifi	Kintampo	Overall unpooled nonadherence	Partially pooled nonadherence^a^
Self‐reported nonadherence	73/157 (46.5%)	123/264 (46.6%)	89/113 (78.8%)	252/502 (50.2%)	234/267 (87.6%)	771/1,303 (59.2%)	65.1% (95% CI 45.0–79.5%)
Detectable levels nonadherence	100/157 (63.7%)	162/264 (61.4%)	77/113 (68.1%)	313/502 (62.4%)	169/267 (63.3%)	821/1,303 (63.0%)	63.1% (60.5–65.6%)
Optimal levels nonadherence	116/1,527 (73.9%)	192/264 (72.7%)	102/113(90.3%)	411/502 (81.9%)	204/267 (76.4%)	1,025/1,303 (78.7%)	79.1% (73.3–84.3%)
Sensitivity % (95% CI)^b^	58.0 (47.7–67.8)	67.3 (59.5–74.4)	84.4 (74.4–91.7)	77.3 (72.3–82.8)	91.1 (85.8–94.9)	76.5 (73.4–79.4)	–
Specificity % (95% CI)^c^	73.7 (60.3–84.5)	86.3 (78.0–92.3)	33.3 (18.6–51.0)	94.7 (90.5–97.4)	18.4 (11.3–27.5)	70.3 (66.0–74.4)	–

CI, confidence interval.

a Overall magnitude of nonadherence to antiepileptic drugs for the five sites and the corresponding confidence intervals obtained from a multilevel model that incorporated between‐sites variation.

b Sensitivity of the self‐reported nonadherence to antiepileptic drugs versus detectable levels.

c Specificity of the self‐reported nonadherence to antiepileptic drugs versus detectable levels.

### Magnitude of nonadherence

The overall prevalence of nonadherence across the five sites was 63.1% (95% CI 60.5–65.6%), as measured by detectable AEDs levels, and 79.1% (95% CI 73.3–84.3%), by optimal AEDs levels; self‐reported nontaking of AEDs was 65.1% (95% CI 45.0–79.5%) (Table 1). The prevalence of nonadherence based on optimal AED levels was 84.9% (95% CI 81.7–87.7%) for children and 73.8% (95% CI 70.4–76.9%) for adults. Additional age‐group‐specific nonadherence estimates are summarized in Figs. S1 and S2.

### Heterogeneity of nonadherence

There was considerable heterogeneity in the site‐specific nontaking of AEDs estimates based on optimal levels (ICC = 3.3%) across the five sites and non‐taking of AEDs estimates from self‐reports (ICC = 18.3%) (Fig. S1). Estimates derived from the direct measurements of nontaking of AEDs from the drug levels were more homogeneous, particularly based on detectable levels (ICC = 0%). We found statistically significant differences in nontaking of AEDs estimates among the five sites as measured by optimal levels of AEDs, with the highest nonadherence (90.3%) recorded in Iganga‐Maguye and the lowest (72.7%) in Ifakara (Table 1).

### Relationship of age with nonadherence

We found that nontaking of AEDs (based on self‐reports and detectable and optimal levels of AEDs) decreased with age, being smallest in those aged 18–28 years (Fig. S2). Univariable association showed that children (<18 years old) had significantly higher nontaking of AEDs estimates than adults as measured by detectable (OR = 1.60, 95% CI 1.28–2.00, p < 0.001) and optimal AEDs levels (OR = 1.96, 95% CI 1.44–2.55, p < 0.001) and by the self‐reported nontaking (OR = 2.00, 95% CI 1.56–2.53, p < 0.001).

### Factors that differed between children and adults with suboptimal AEDs

Among those without optimal AED levels, fewer children were found to have burn marks (OR = 0.41, 95% CI 0.28–0.63, p < 0.001) and learning difficulties (OR = 0.63, 95% CI 0.44–0.89, p = 0.009) than adults. More children than adults had a history of status epilepticus (OR = 1.98, 95% CI 1.40–2.88, p < 0.001) (Table S6).

### Factors associated with nonadherence in children

Several factors were investigated for an association with nontaking of AEDs as measured from the optimal levels in the univariable multilevel logistic regression model (Table 2). From the likelihood ratio test, there was no evidence that the investigated variables influenced nontaking of AEDs differently across the five sites (all p values were > 0.05).

**Table 2:** Univariable analysis of factors associated with nonadherence to antiepileptic drugs in children^a^

Factors	Adhering to medication n = 86	Not adhering to medication n = 484	Odds ratio (95% CI)	p value
Age (years): median (IQR)	12.0 (8.25–14.23)	11.0 (7.0–14.9)	0.97 (0.92–1.01)	0.20
Sex				
Male	43 (50.0%)	222 (45.9%)	0.85 (0.52–1.38)	0.48
Mother's religious affiliation				
Christianity	52 (69.3%)	278 (67.0%)	1	
Islam	16 (21.3%)	110 (26.5%)	1.29 (0.69–2.52)	0.41
Traditionalist	7 (9.3%)	27 (6.5%)	0.72 (0.29–2.07)	0.47
Mother's marital status				
Married	65 (75.6%)	375 (77.5%)	1	
Single/separated/divorced/widowed	21 (24.4%)	109 (22.5%)	0.90 (0.52–1.62)	0.70
Mother's education level				
Postprimary	6 (16.2%)	47 (21.1%)	1	
≤Primary school	31 (83.8%)	176 (78.9%)	0.70 (0.23–1.91)	0.50
Mother's occupation				
Employed	5(10.9%)	33 (12.8%)	1	
Unemployed	41 (89.1%)	224 (87.2%)	0.83 (0.24–2.31)	0.71
Father's education level				
Postprimary	14 (29.2%)	77 (28.8%)	1	
≤Primary school level	34 (70.8%)	190 (71.2%)	1.02 (0.48–2.07)	0.96
Father's occupation				
Employed	12 (27.3%)	86 (29.0%)	1	
Unemployed	32 (72.7%)	211 (71.0%)	0.92 (0.41–1.94)	0.82
Mother's age at first birth				
≥18 years	38 (65.5%)	201 (57.4%)	1	
<18 years	20 (34.5%)	149 (42.6%)	1.41 (0.76–2.66)	0.25
Sibling has seizures				
No	12 (14.0%)	52 (10.7%)	1	
Yes	74 (86.0%)	432 (89.3%)	1.35 (0.62–2.71)	0.39
Snores more than 3 days per week				
No	36 (45.0%)	206 (44.0%)	1	
Yes	44 (55.0%)	262 (56.0%)	1.04 (0.63–1.72)	0.87
Place of birth: home				
No	30 (35.3%)	174 (36.4%)	1	
Yes	55 (64.7%)	304 (63.6%)	0.95 (0.57–1.58)	0.84
Burn marks				
No	73 (85.9%)	436 (90.1%)	1	
Yes	12 (14.1%)	48 (9.9%)	0.67 (0.33–1.45)	0.24
Sought traditional medicine				
No	21(26.9%)	140 (31.7%)	1	
Yes	57(73.1%)	302 (68.3%)	0.79 (0.44–1.39)	0.40
Previous hospitalization				
No	34 (40.0)	267 (55.5%)	1	
Yes	51(60.0%)	214 (44.5%)	0.53 (0.32–0.88)	0.008
Learning difficulties				
No	59 (69.4%)	402 (83.1%)	1	
Yes	26 (30.6%)	82 (16.9%)	0.46 (0.27>–0.81)	0.003
Neurological deficits				
No	74 (87.1%)	410 (86.8%)	1	
Yes	11 (12.9%)	64 (13.2%)	1.05 (0.52–2.31)	0.89
Frequent seizures				
No	70 (81.4%)	416 (86.1%)	1	
Yes	16 (18.6%)	67 (13.9%)	0.70 (0.38–1.38)	0.25
Focal seizures				
No	43 (50.0%)	259 (53.5%)	1	
Yes	43 (50.0%)	225 (46.5%)	0.87 (0.53–1.41)	0.55
Status epilepticus				
No	59 (73.8%)	304 (68.6%)	1	
Yes	21 (26.2%)	139 (31.4%)	1.28 (0.73–2.32)	0.36

CI, confidence interval; IQR, interquartile range.

a Nonadherence to antiepileptic drugs was evaluated on the basis of optimal drug levels in the blood.

The four variables that had a univariable p value ≤0.25 (age, mother's age at first birth, previous hospitalization, and learning difficulties) were used to build a multivariable model. Nontaking of AEDs was independently associated with history of previous hospitalization (OR = 0.50, 95% CI 0.28–0.87) and presence of learning difficulties (OR = 0.51, 95% CI 0.26–0.97) (Table 3).

**Table 3:** Multivariable analysis for factors associated with nonadherence to antiepileptic drugs as measured by optimal levels in the blood of children and adults

	Odds ratio (95% CI)	p value
Children		
Age (years)	0.90 (0.96–1.02)	0.17
Mother's age at first birth	1.35 (0.74–2.44)	0.35
Previous hospitalization		
No	1	
Yes	0.50 (0.28–0.87)	0.02
Learning difficulties		
No	1	
Yes	0.51 (0.26–0.97)	0.04
Adults		
Age (years)	1.00 (0.98–1.02)	0.88
Marital status		
Married	1	
Single/separated/divorced/widowed	0.84 (0.44–1.60)	0.59
Level of education		
Postprimary	1	
≤Primary school	1.48 (0.88–2.56)	0.14
Place of birth: home		
No	1	
Yes	2.06 (1.21–3.61)	0.01
Snores more than three times per week		
No	1	
Yes	0.76 (0.44–1.24)	0.26
Burn marks		
No	1	
Yes	0.53 (0.29–0.96)	0.04
Sought traditional medicine		
No	1	
Yes	0.37 (0.18–0.79)	0.006
Previous hospitalization		
No	1	
Yes	0.84 (0.48–1.38)	0.48
Learning difficulties		
No	1	
Yes	0.88 (0.52–1.65)	0.71

CI, confidence interval.

### Factors associated with nonadherence in adults

Several factors were investigated for an association with nontaking of AEDs as measured from the optimal levels in the univariable multilevel logistic regression model (Table 4). From the likelihood ratio test, there was also no evidence that the variables under consideration influence the nontaking of AEDs differently across the sites (all p values were > 0.05).

**Table 4:** Univariable analysis for factors associated with nonadherence to antiepileptic drugs in adults^a^

Factors	Adhering to medication n = 192	Not adhering to medication n = 541	Odds ratio (95% CI)	p value
Age (years): median (IQR)	27.8 (22.9–38.0)	29.5 (23.0–41.0)	1.01 (0.99–1.02)	0.14
Sex: Female	95 (49.5%)	272 (50.3%)	1.03 (0.73–1.46)	0.85
Religion				
Christianity	118 (75.2%)	322 (75.2%)	1	
Islam	25 (15.9%)	70(16.4%)	1.02 (0.61–1.77)	0.92
Traditionalist	14 (8.9%)	36 (8.4%)	0.94 (0.48–1.96)	0.85
Marital status				
Married	50 (26.5%)	172 (33.5%)	1	
Single/separated/divorced/widowed	136 (73.5%)	341 (66.5%)	0.73 (0.49–1.07)	0.09
Level of education				
Postprimary	46 (42.2%)	105 (35.4%)	1	
≤Primary school	63 (57.8%)	192 (64.6%)	1.34 (0.83–2.14)	0.21
Occupation				
Employed	6 (8.6%)	14 (7.9%)	1	
Unemployed	64 (91.4%)	163 (92.1%)	1.09 (0.33–3.19)	0.86
Sibling has seizures				
No	166 (86.5%)	484 (89.5%)	1	
Yes	26 (13.5%)	57 (10.5%)	0.75 (0.45–1.29)	0.26
Snores more than three days per week				
No	75 (39.9%)	245 (48.7%)	1	
Yes	113 (60.1%)	258 (51.3%)	0.70 (0.49–0.99)	0.04
Place of birth: home				
No	62 (33.7%)	134 (25.7%)	1	
Yes	122 (66.3%)	388 (74.3%)	1.47 (1.00–2.15)	0.04
Burn marks				
No	134 (69.8%)	421 (78.1%)	1	
Yes	58 (30.2%)	118 (21.9%)	0.65 (0.44–0.96)	0.02
Sought traditional medicine				
No	27 (15.1%)	117 (23.3%)	1	
Yes	152 (84.9%)	385 (76.7%)	0.58 (0.35–0.94)	0.02
Previous hospitalization				
No	104 (54.2%)	320 (59.7%)	1	
Yes	88 (45.8%)	216 (40.3%)	0.80 (0.56–1.13)	0.18
Learning difficulties				
No	135 (70.3%)	400 (74.3%)	1	
Yes	57 (29.7%)	138 (25.7%)	0.82 (0.56–1.20)	0.28
Neurological deficits				
No	167 (87.0%)	462 (85.7%)	1	
Yes	25 (13.0%)	77 (14.3%)	1.11 (0.67–1.89)	0.66
Frequent seizures				
No	175 (91.1%)	480 (88.7%)	1	
Yes	17 (8.9%)	61 (11.3%)	1.31 (0.73–2.46)	0.35
Focal seizures				
No	96 (50.0%)	269 (49.7%)	1	
Yes	96 (50.0%)	272 (50.3%)	1.01 (0.72–1.42)	0.95
Status epilepticus				
No	148 (84.1%)	393 (80.2%)	1	
Yes	28 (15.9%)	97 (19.8%)	1.36 (0.84–2.26)	0.26

CI, confidence interval; IQR, interquartile range.

a Nonadherence to antiepileptic drugs as evaluated on the basis of optimal drug levels in the blood.

Eight factors (Table 4) with univariable p values ≤ 0.25 were used to build a multivariable model. Out of these factors, being born at home (OR = 2.06, 95% CI 1.21–3.61), presence of burn marks (OR = 0.53, 95% CI 0.29–0.96), and seeking traditional medicine (OR = 0.37, 95% CI 0.18–0.79), were independently associated with the nontaking of AEDs based on optimal levels (Table 3).

## Discussion

We estimated the prevalence of nonadherence at 63% on the basis of detectable AED levels and 79% on the basis of optimum levels across five rural sites in Africa. Nonadherence as measured by AED levels was more homogeneous across the sites than self‐reported nonadherence, suggesting that AED levels detectable in blood are more reliable. Also, social desirability bias (a response bias in which respondents tend to give responses that are favorable or acceptable by others) linked to stigma may prompt persons with epilepsy in Africa not to disclose their nonadherence to AEDs. The differences observed in nonadherence determined by optimal levels may be explained by differences in specific AEDs prescribed at the sites whose pharmacokinetics and pharmacodynamics differ, although heterogeneity owing to other site‐specific factors cannot be excluded. Estimates for nonadherence were significantly greater in children.

### Sensitivity and specificity

The sensitivity and specificity of the self‐reported nonadherence compared to detectable levels varied with sites. In Kilifi, the sensitivity and specificity of self‐reporting (measured with the Morisky scale [a four‐item questionnaire]) compared to detectable levels were lower than those reported in this study, where self‐reported nonadherence was based on one question.^13^ This suggests that the Morisky scale is stringent and that one‐item self‐reported adherence is a better correlate of detectable AEDs levels. In fact, nonadherence from self‐report and that from detectable levels were comparable. The sensitivity and specificity, however, varied across the sites, with some sites registering low values, suggesting that self‐reported nontaking is unreliable and can be influenced by different factors within each site.

### Magnitude of nonadherence

Estimates of nonadherence as measured by detectable levels (63%) and by self‐reports documented in this study are similar to those previously reported in Kenyan studies and in a recent systematic review.^1^, ^13^, ^15^ Overall nonadherence is in line with the results of a recent review, which estimated nonadherence for detectable levels at 59% (95% CI 32–85%).^25^ Nonadherence estimates as measured by optimum drug levels, however, were greater (79%), probably because these measures were based on higher cut‐off AED levels that are clinically beneficial. These findings highlight nonadherence as a widespread problem in sub‐Saharan Africa, and this in part explains the large treatment gap in Africa. It is likely that some common causes of nonadherence do exist in these countries and could include cost of AEDs, distance to health facilities, and cultural beliefs about epilepsy and treatment.^8^, ^14^ Some of these specific causes were not investigated in this study, and future studies at each site are warranted.

There was heterogeneity in rates of nonadherence to AEDs as measured from blood levels and self‐reports, although it was significantly greater in the latter than in the former. Heterogeneity in observed nonadherence may be related to the social desirability bias and epilepsy stigma (for self‐reported nonadherence) and methodological differences (for nonadherence measured by blood levels). We, however, attempted to use standardized questionnaires and procedures for blood collection and transportation to minimize heterogeneity.

### Nonadherence and its association with age

We found that nonadherence is greater in children, as in previous reports.^13^, ^15^ The reasons for this are not yet clear but may be related to health‐seeking behaviors or less severe epilepsy, as suggested by the presence of burn marks (though not statistically significant) and history of previous hospitalization for any condition, including febrile illnesses. Epilepsy may be perceived as severe and requiring prompt treatment if it is associated with burn marks or learning disability, particularly in children.^5^ Previous hospitalization is associated with AED taking, probably because it suggests positive health‐seeking behavior or was documented in those living near hospitals.^3^

The high nonadherence estimates in children could also be attributed to the fact that collection and taking of medication depend on adults, who may fail to seek treatment for their children, possibly owing to cultural beliefs.^25^ Furthermore parents may think that their child has acute febrile seizures, which are not considered serious, and therefore they are not motivated to seek treatment for their child.^26^ Differences in pharmacokinetics of drugs in these two groups—children usually have faster drug elimination rates and reduced blood half‐lives compared to adults—may affect our estimates.^27^ A titration schedule that ensures a maximum tolerated dose should be explored to ensure optimum levels for seizure control in children.^27^ Bimodal age‐related incidence peak of status epilepticus is present in the very young and very old in populations; thus, the low nonadherence in children with status epilepticus could suggest this is a biological phenomenon and not the result of AED adherence. Whatever the reason, the large nonadherence estimates in African children need to be addressed because (1) most complications, including those that are potentially fatal such as convulsive status epilepticus, were more common in this group;^18^, ^28^ and (2) children with epilepsy have a poor quality of life, which could hinder them from attaining full developmental potential, making this group of special clinical importance.^29^, ^30^

### Factors associated with nonadherence to treatment

Nonadherence was associated with history of previous hospitalization in children, a factor that may be a surrogate marker of health‐seeking behavior or distance to health facilities.^3^ In a recent Kenyan study, previous hospitalization was independently associated with admission to hospital for epilepsy, suggesting that distance influences the decision to seek biomedical treatment for epilepsy in this area.^3^ Alternatively, those without previous hospitalization may be people who do prefer not to access biomedical services, thereby the association with nonadherence.

In adults, nonadherence was associated with being born at home, which could suggest negative attitudes toward use of biomedical facilities. This is supported by the association between seeking traditional treatment and taking of AEDs, whereby people who visit traditional healers are now likely to go to the hospital following educational interventions in this area.^15^ The association between traditional medicine use and the taking of AEDs is interesting and should be interpreted in the contexts of two groups of people with epilepsy. The first group exclusively prefers traditional medicine to biomedical treatment and would not resort to the latter even when the former fails, thereby contributing to the large treatment gap for epilepsy. The second group uses both traditional and biomedical treatments, in either order and concurrently, and may therefore appear to be adhering to antiepileptic drugs, as supported by results from this multisite study. This study found a strong association between presence of burn marks and the taking of AEDs. Burns are often caused by accidents during seizures and may prompt individuals to seek biomedical treatment for their epilepsy.^13^

### Strengths and limitations

We used a standard methodology to identify people with epilepsy and to determine nonadherence estimates across five sites.^5^, ^17^ We also used a robust statistical approach that accounted for possible clustering within the sites. The sample size was large enough to allow for adequate power to measure differences between groups. The limitation is that we were not able to investigate known causes of nonadherence in all sites identified from other studies. The other limitation is the selection bias with overrepresentation of people with focal seizures (Table S2), which may be due to epilepsy stigma or misperception of epilepsy as less severe.^31^ Only active convulsive epilepsy was included in the study, and nonadherence may be different in those with inactive epilepsy and nonconvulsive epilepsy not in the study. Additionally, the assigned daily dose was unknown, and we could only speculate that the patient received (and took) a correct dose at the discretion of the caring physician.

## Conclusion

Many people with epilepsy in Africa do not take AEDs (about 80%, based on optimum levels), and this nonadherence is worse in children, in whom dosing schedules should be closely monitored by clinicians to ensure maximum tolerated and effective doses and AED taking should be supervised by parents. The reasons for the significantly higher nonadherence estimates found in children compared with adults warrant further studies in sub‐Saharan Africa. The high rate of nontaking of AEDs in children needs to be addressed because most complications such as convulsive status epilepticus were found to be more common in this group and are associated with significant mortality and neurological damage. Untreated epilepsy can hinder children from attaining their full developmental and societal potential.

## Disclosure

None of the authors has any conflict of interest to disclose in relation to this work. We confirm that we have read the Journal's position on issues involved in ethical publication and affirm that this report is consistent with those guidelines.

## Supporting information


**Appendix S1.** The SEEDS writing group.
**Table S1.** Factors investigated for an association with the epilepsy treatment gap in children and adults separately.
**Table S2.** Comparison of the characteristics of the people with epilepsy who gave versus those who did not give blood.
**Table S3.** Characteristics of the study participants: children.
**Table S4.** Characteristics of the study participants: adults.
**Table S5.** Cross‐classification of the reported AEDs and the detected AEDs among the people with epilepsy who reported taking AEDs.
**Table S6.** Characteristics associated with being a child among people with epilepsy who did not have optimal levels of AEDs in the blood.
**Figure S1.** Magnitude of site‐specific nonadherence to AEDs.
**Figure S2.** Magnitude of age‐specific nonadherence to AEDs.
