# Supplementary material for: Magnitude and factors associated with nonadherence to antiepileptic drug treatment in Africa: A cross‐sectional multisite study
Source: Epilepsia Open. 2017 Mar 30;2(2):226–35. doi: 10.1002/epi4.12052 (PMC5719857; doi:10.1002/epi4.12052)
Supplement: Supplementary file 1 — Appendix S1. The SEEDS writing group. Table S1. Factors investigated for an association with the epilepsy treatment gap in children and adults separately. Table S2. Comparison of the characteristics of the people with epilepsy who gave versus those who did not give blood. Table S3. Characteristics of the study participants: children. Table S4. Characteristics of the study participants: adults. Table S5. Cross‐classification of the reported AEDs and the detected AEDs among the people with epilepsy who reported taking AEDs. Table S6. Characteristics associated with being a child among people with epilepsy who did not have optimal levels of AEDs in the blood. Figure S1. Magnitude of site‐specific nonadherence to AEDs. Figure S2. Magnitude of age‐specific nonadherence to AEDs. [file EPI4-2-226-s001.pdf]

Supplementary file 1: The SEEDS writing group

Agincourt HDSS, South Africa: Ryan Wagner, Rhian Twine, Myles Connor, F Xavier Gómez Olivé, Mark Collinson, Kathleen Kahn, Stephen Tollman; ***Ifakara HDSS, Tanzania:*** Honratio Masanja, Alexander Mathew (deceased); ***Iganga-Mayuge HDSS, Uganda:*** Angelina Kakooza, George Pariyo, Stefan Peterson, Donald Ndyomughenyi; ***Kilifi HDSS, Kenya:*** Fredrick Ibinda (deceased), Symon M Kariuki, Anthony K Ngugi, Rachael Odhiambo, Eddie Chengo, Martin Chabi, Evasius Bauni, Gathoni Kamuyu, Victor Mung'ala Odera (deceased), James O Mageto, Charles R Newton; ***Kintampo HDSS, Ghana:*** Ken Ae-Ngibise, Bright Akpalu, Albert Akpalu, Francis Agbokey, Patrick Adjei, Seth Owusu-Agyei; ***London School of Hygiene and Tropical Medicine, UK:*** Christian Bottomley, Immo Kleinschmidt; ***King's College London, UK:*** Victor C K Doku; ***Swiss Tropical Institute, Switzerland:*** Peter Odermatt; ***University College London, UK:*** Brian Neville (deceased), Josemir W Sander, Steve White; ***National Institutes of Health, USA:*** Thomas Nutman; ***Centers for Disease Control and Prevention, USA:*** Patricia Wilkins, John Noh.

Supplementary file 2: Factors investigated for an association with the epilepsy treatment gap in children and adults separately

Children	Adults
Age	Age
Sex	Sex
Mothers religious affiliation	Religion
Mothers marital status	Marital status
Mothers education level	Level of education
Mothers occupation	Occupation
Fathers education level	-
Fathers occupation	-
Mothers age at first birth	-
Sibling has seizures	Sibling has seizures
Snores more than three days per week	Snores more than three days per week
Place of birth (Home/hospital)	Place of birth (Home/hospital)
Presence of burn marks	Presence of burn marks
Sought traditional medicine	Sought traditional medicine
Previous hospitalisation	Previous hospitalisation
Learning difficulties	Learning difficulties
Neurological deficits	Neurological deficits
Frequent seizures	Frequent seizures
Focal seizures	Focal seizures
Status epilepticus	Status epilepticus

Supplementary file 3: Comparison of the characteristics of the people with epilepsy who gave versus those who did not give blood.

			Univariate analysis		Multivariable analysis*	
**Characteristics**	**Sample assayed n=1303**	**Sample not assayed n=888**	**Odds ratio (95%CI)**	**p-vale**	**Odds ratio (95%CI)**	**p-vale**
**Sex: female**	672 (51.6%)	454 (51.1%)	1.02 (0.86-1.21)	0.84	-	-
**Sibling has seizures**						
No	1156 (88.7%)	811 (91.3%)	1		1.12 (0.77-1.64)	
Yes	147 (11.3%)	77 (8.7%)	1.34 (0.99-1.81)	0.05	1	0.56
**Snores more than three nights a week**						
No	562 (45.4%)	415 (49.5%)	1		1	
Yes	677 (54.6%)	424 (50.5%)	1.18 (0.99-1.41)	0.07	1.04 (0.82-1.35)	0.68
**Place of birth: home**						
No	400 (31.5%)	365 (43.3%)	1		1	
Yes	869 (68.5%)	477 (56.7%)	1.66 (1.38-2.00)	<0.001	1.15 (0.88-1.55)	0.35
**Burn marks**						
No	1064 (81.8%)	776 (87.4%)	1		1	
Yes	236 (18.2%)	112 (12.6%)	1.54 (1.20-1.98)	<0.001	1.17 (0.84-1.66)	0.36
**Sought traditional medicine**						
No	305 (25.4%)	258 (33.0%)	1		1	
Yes	896 (74.6%)	525 (67.0%)	1.44 (1.18-1.77)	<0.001	1.06 (0.79-1.43)	0.71
**Previous hospitalisation**						
No	725 (56.0%)	512 (58.0%)	1			
Yes	569 (44.0%)	370 (42.0%)	1.09 (0.91-1.30)	0.35	-	-
**Learning difficulties**						
No	996 (76.7%)	691 (77.8%)	1		-	-
Yes	303 (23.3%)	197 (22.2%)	1.06 (0.86-1.32)	0.94		
**Neurological deficits**						
No	1123 (86.4%)	745 (83.9%)	1			
Yes	177 (13.6%)	143 (16.1%)	0.82 (0.64-1.05)	0.11	**0.70 (0.50-0.98)**	**0.03**
**Frequent seizures**						
No	571 (67.0%)	329 (62.9%)	1			
Yes	281 (33.0%)	194 (37.1%)	0.83 (0.66-1.06)	0.12	0.84 (0.65-1.09)	0.22
**Focal seizures**						
No	667 (51.2%)	543 (61.1%)	1			
Yes	636 (48.8%)	345 (38.9%)	1.50 (1.26-1.79)	<0.001	**1.38 (1.07-1.75)**	**0.02**
**Status epilepticus**						
No	905 (76.1%)	525 (73.9%)	1			
Yes	284 (23.9%)	185 (26.1%)	0.89 (0.72-1.11)	0.29		

*Multivariable analysis is adjusted for being a child.

Supplementary file 4: Characteristics of the study participants: children

Characteristics	Agincourt n=47	Ifakara n=131	Iganga-Maguye n=75	Kintampo n=88	All five sites combined n=570	p value‡
**Childs age (median (IQR)**	12.0 (7.0-14.5)	11.6 (7.3-15.0)	7.0 (5.0-12.0)	13.0 (10.8-15.0)	11.0 (7.0-14.8)	<0.001
**Sex :**Female	27 (57.4%)	64 (48.9%)	39 (52.0%)	51 (58.0%)	305 (53.5.0%)	0.69
**Mothers marital status**						
Married	18 (38.3%)	99 (75.6%)	66 (88.0%)	70 (79.5%)	440 (77.2%)	
Single/separated/divorced/windowed	29 (61.7%)	32 (24.4%)	9 (12.0%)	18 (20.5%)	130 (22.8%)	<0.001
**Mothers education level**						
Post primary	19 (57.6%)	1(1.4%)	14 (37.8%)	14 (66.7%)	207 (79.6%)	
≤ Primary school	14 (42.4%)	72 (98.6%)	23 (62.2%)	7 (33.3%)	53 (20.4%)	<0.001*
**Mother's religious affiliation**						
Christianity	12 (100%)	94 (76.4%)	32 (42.7%)	48 (69.6%)	330 (67.3%)	
Islam	0	29 (23.6%)	43 (57.3%)	21 (30.4%)	126 (25.7%)	
Traditionalist	0	0	0	0	34 (6.9%)	<0.001*
**Mother's occupation**						
Employed	2 (28.6%)	2 (2.2%)	7 (31.8%)	3 (4.8%)	38 (12.5%)	
Unemployed	5 (71.4%)	89 (97.8%)	15 (68.2%)	59 (95.2%)	265 (87.5%)	<0.001*
**Father's education level**						
≤ Primary school level	13 (50.0%)	69 (97.2%)	23 (76.7%)	10 (26.3%)	224 (71.1%)	
Post primary	13 (50.0%)	2 (2.8%)	7 (23.3%)	28 (73.7%)	91 (28.9%)	<0.001
**Father's occupation**						
Employed	9 (64.3%)	6 (8.1%)	16 (47.1%)	4 (6.0%)	98 (28.7%)	
Unemployed	5 (35.7%)	68 (91.9%)	18 (52.9%)	63 (94.0%)	243(71.3%)	<0.001*
**Mothers age at first birth**						
< 18 years	11 (28.2%)	26 (32.9%)	25 (52.1%)	41 (52.6%)	169 (41.4%)	
≥ 18 years	28 (71.8%)	53 (67.1%)	23 (47.9%)	37 (47.4%)	239 (58.6%)	0.02
**Sibling has seizures**						
Yes	43 (91.5%)	123 (93.9%)	64 (85.3%)	65 (73.9%)	506 (88.8%)	
No	4 (8.5%)	8 (6.1%)	11(14.7%)	23 (26.1%)	64 (11.2%)	<0.001*
**Place of birth: home**						
Yes	12 (26.1%)	52(40.9%)	23 (30.7%)	73 (83.9%)	359 (63.8%)	
No	34 (73.9%)	75 (59.1%)	52 (69.3%)	14 (16.1%)	204 (36.2%)	<0.001
**Burn marks**						
Yes	1 (2.1%)	14 (10.7%)	1(1.3%)	8 (9.2%)	60 (10.5%)	
No	46 (97.9%)	117 (89.3%)	74 (98.7%)	79 (90.8%)	509 (89.5%)	0.001*
**Sought traditional medicine**						
Yes	20 (71.4%)	87(77.0%)	37 (52.1%)	63 (75.0%)	359 (69.0%)	
No	8 (28.6%)	26 (23.0%)	34 (47.9%)	21 (25.0%)	161 (31.0%)	0.006
**Previous hospitalisation**						

Yes	6 (13.0%)	68 (52.3%)	5 (6.7%)	36 (41.4%)	265 (46.8%)	
No	40 (87.0%)	62 (47.7%)	70 (93.7%)	51 (58.6%)	301 (53.2%)	<0.001
**Learning difficulties**						
Yes	7 (14.9%)	15 (11.5%)	10 (13.3%)	19 (21.8%)	108 (19.0%)	
No	40 (85.1%)	116 (88.5%)	65 (86.7%)	68 (78.2%)	461 (81.0%)	0.01
**Neurological deficits**						
Yes	4 (8.5%)	11 (8.4%)	10 (13.3%)	10 (11.5%)	75 (13.2%)	
No	43 (91.5%)	120 (91.6%)	65 (86.7%)	77 (88.5%)	494 (86.8%)	0.12
**Frequent seizures**						
No	44 (95.7%)	113 (86.3%)	65 (86.7%)	72 (81.8%)	486 (85.4%)	
Yes	2 (4.3%)	18 (13.7%)	10 (13.3%)	16 (18.2%)	83 (14.6%)	0.001
**Focal seizures**						
No	34 (72.3%)	85 (64.9%)	53 (70.7%)	63 (71.6%)	302 (53.0%)	
Yes	13 (27.7%)	46 (35.1%)	22 (29.3%)	25 (28.4%)	268 (47.0%)	0.04
**Status epilepticus**						
No	28 (65.1%)	97 (92.4%)	43 (59.7%)	71 (87.7%)	363 (69.4%)	
Yes	15 (34.9%)	8 (7.6%)	29 (40.3%)	10 (12.3%)	160 (30.6%)	0.39

‡ p values for differences in distribution of characteristics across the five sites; ***** implies that a Fishers exact test was performed.

Supplementary file 5: Characteristics of the study participants: adults

Characteristics	Agincourt n=110	Ifakara n=133	Iganga-Maguye n=38	Kintampo n=179	All five sites combined† n=733	p value‡
**Age (years):** median (IQR)	38.0 (27.0-47.0)	29.7 (23.2-40.5)	30.5 (22.0-43.0)	26.0 (21.0-34.0)	29.0 (23.0-40.0)	<0.001
**Sex :** Female	60 (54.5)	58 (43.6%)	20 (52.6)	98 (54.7)	367 (50.1%)	0.26
**Religion**						
Christianity	25 (100%)	100 (80.0%)	25 (71.4%)	115 (75.2%)	440 (75.2%)	
Islam	0	23 (18.4%)	10 (28.6%)	34 (22.2%)	95 (16.2%)	
Traditionalist	0	2 (1.6%)	0	4 (2.6%)	50 (8.5%)	<0.001*
**Marital status**						
Married	30 (27.3%)	53 (40.8%)	12 (34.3%)	44 (25.3%)	222 (31.6%)	
Single/separated/divorced/windowed	80 (72.7%)	77 (59.2%)	23 (65.7%)	130 (74.7%)	480 (68.4%)	0.05
**Level of education**						
≤ Primary school	49 (62.0%)	64 (97.0%)	12 (52.2%)	6 (5.8%)	255 (62.8%)	
Post primary	30 (38.0%)	2 (3.0%)	11 (47.8%)	98 (94.2%)	151 (37.2%)	<0.001
**Occupation**						
Employed	2 (66.7%)	0	2 (25.0%)	1 (1.4%)	20 (8.1%)	
Unemployed	1 (33.3%)	75 (100%)	6 (75.0%)	71 (98.6%)	227 (92.9%)	<0.001*
**Sibling has seizures**						
Yes	5 (4.5%)	20 (15.0%)	4 (10.5%)	37 (20.7%)	83 (11.3%)	
No	105 (95.5%)	113(85.0%)	34(89.5%)	142 (79.3%)	650 (88.7%)	<0.001*
**Place of birth: home**						
Yes	52 (49.0%)	75 (64.1%)	14 (36.8%)	154 (87.5%)	510 (72.2%)	
No	50 (51.0%)	42 (35.9%)	24 (63.2%)	22 (12.5%)	196 (27.8%)	<0.001
**Burn marks**						
Yes	25 (22.7%)	28 (21.1%)	6 (15.8%)	38 (21.5%)	176 (24.1%)	
No	85 (77.3%)	105 (78.9%)	32 (84.2%)	139 (78.5%)	555 (75.9%)	0.17
**Sought traditional medicine**						
Yes	60 (67.7%)	100 (84.7%)	24 (63.2%)	141 (83.9%)	537 (78.9%)	
No	30 (33.3%)	18 (15.3%)	14 (36.8%)	27 (16.1%)	144 (21.1%)	0.001
**Previous hospitalization**						
Yes	25 (22.9%)	55 (41.7%)	5 (13.2%)	63 (35.2%)	304 (41.8%)	
No	84 (77.1%)	77 (58.3%)	33 (86.8%)	116 (64.8%)	424 (58.2%)	<0.001
**Learning difficulties**						
Yes	37 (33.6%)	15 (11.3%)	10 (26.3%)	54 (30.7%)	195 (26.7%)	
No	73 (66.4%)	118 (88.7%)	28 (73.7%)	122 (69.3%)	535 (73.3%)	<0.001
**Neurological deficits**						
Yes	25 (22.7%)	7 (5.3%)	9 (23.7%)	17 (9.6%)	102 (14.0%)	
No	85 (77.3%)	126 (94.7%)	29 (76.3%)	160 (90.4%)	629 (86.0%)	<0.001
**Frequent seizures**						
Yes	7 (6.4%)	14(10.5%)	3 (7.9%)	20 (11.2%)	78 (10.6)	

No	103 (93.6%)	119 (89.5%)	38 (92.1%)	159 (88.8%)	655 (89.4%)	0.51*
**Focal seizures**						
Yes	55 (50.0%)	40 (30.1%)	18 (47.4%)	69 (38.5%)	368 (50.2%)	
No	55 (50.0%)	93 (69.9%)	20 (52.6%)	110 (61.5%)	365 (49.8%)	<0.001
**Status epilepticus**						
Yes	21 (21.4%)	2 (2.1%)	17 (48.6%)	9 (5.3%)	21 (21.4%)	
No	77 (78.6%)	93 (97.9%)	18 (51.4%)	162 (94.7%)	77 (78.6%)	<0.001

‡ p values for differences in distribution of characteristics across the five sites; ***** implies that a Fishers exact test was performed.

Supplementary file 6: Cross classification of the reported AEDs and the detected AEDS among the PWE who reported taking AEDs

AED reported	AED detected						
	PB only	PH only	CB only	PB & PH	PB & CB	PH & CB	PB, PH & CB
Phenobarbital (PB) only	134	3	1	25	0	1	3
Phenytoin (PH) only	0	8	0	0	0	0	0
Carbamazepine (CB) only	2	1	8	0	0	4	0
PB & PH	22	6	0	24	2	1	1
PB & CB	10	1	6	0	7	0	7
PH & CB	0	0	0	0	0	1	0

Supplementary file 7: Magnitude of site specific non-adherence to AEDs

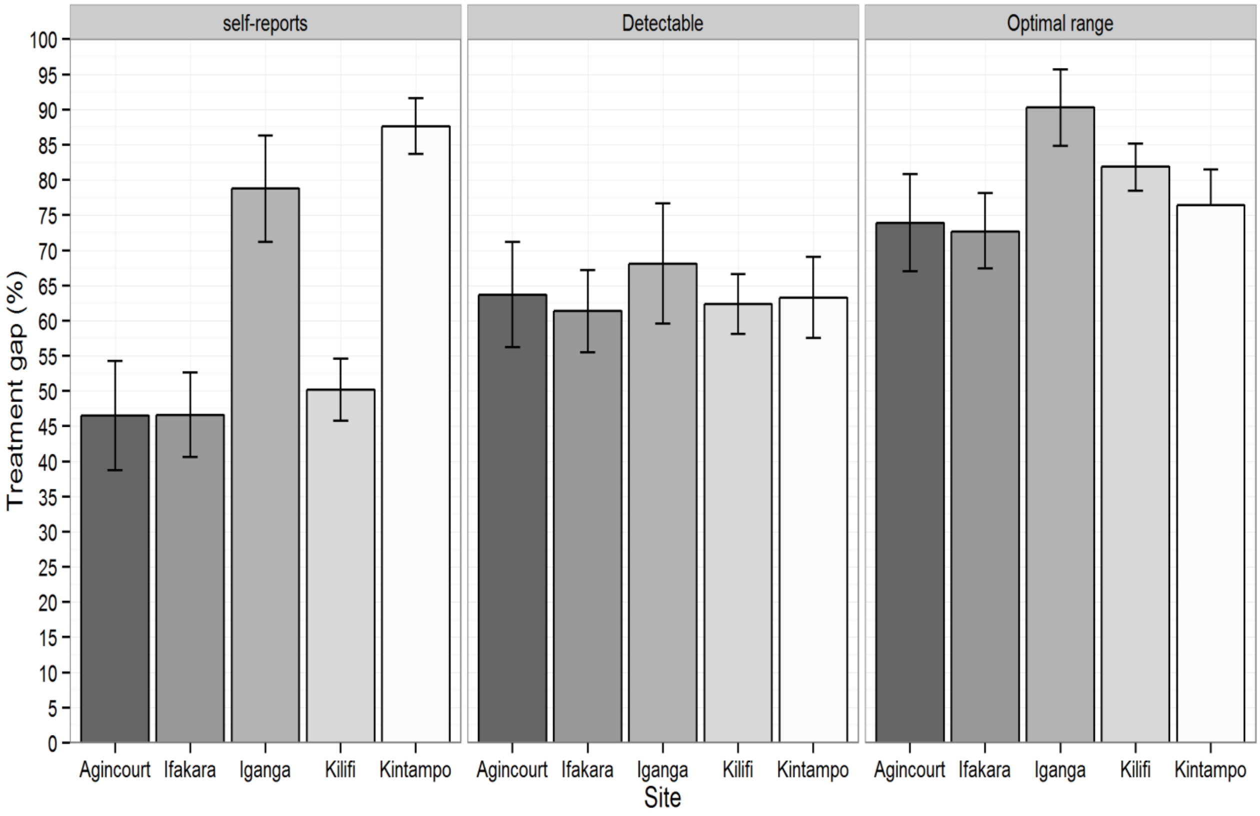

A graphical representation of the non-adherence to AEDs for each of the five sites based on self-reports, and as measured by detectable and optimal levels of anti-epileptic drugs in the blood.

Supplementary file 8: Magnitude of age specific non-adherence of AEDs

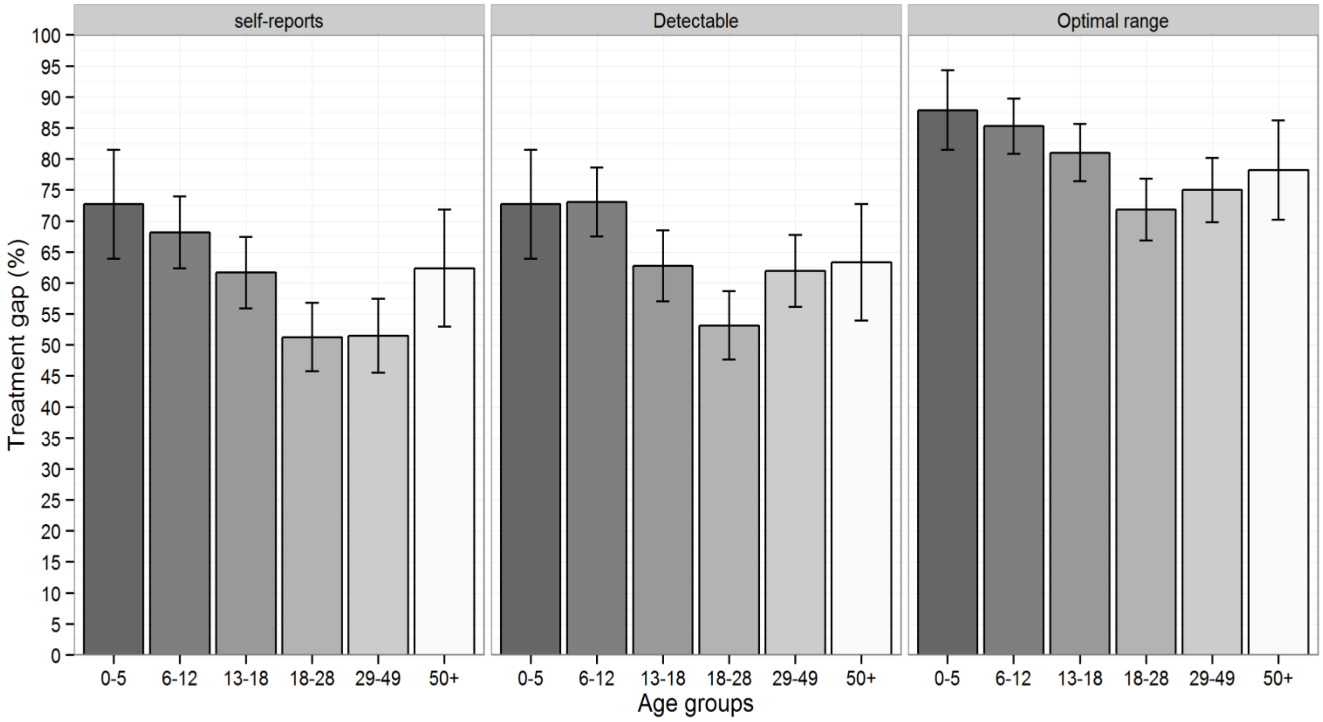

A graphical representation of the non-adherence to AEDs by age groups based on self-reports, and as measured by detectable and optimal levels of anti-epileptic drugs in the blood.

Supplementary file 9: Characteristics associated with being a child among people with epilepsy who did not have optimal levels of anti-epileptic drugs in blood.

Characteristics		Univariable analysis			Multivariable analysis	
	**Children (n=484)**	**Adults (n=541)**	**Odds ratio (95%CI)**	**p value**	**Odds ratio (95%CI)**	**p value**
**Sex: female**	262 (54.1%)	272 (50.3%)	1.23 (0.95-1.56)	**0.12**	1.06 (0.79-1.45)	0.62
**Sibling has seizures**						
No	432 (89.3%)	484 (89.5%)	1			
Yes	52 (10.7%)	57 (10.5%)	1.10 (0.75-1.62)	0.69	-	-
**Place of birth: home**						
No	174 (36.4%)	134 (25.7%)	1		1	
Yes	304 (63.6%)	388 (74.3%)	0.68 (0.49-0.92)	**0.01**	0.79 (0.55-1.09)	0.16
**Burn marks**						
No	436 (90.1%)	421 (78.1%)	1		1	
Yes	48 (9.9%)	118 (21.9%)	0.39 (0.27-0.57)	**<0.001**	**0.41 (0.28-0.63)**	**<0.001**
**Sought traditional medicine**						
No	140 (31.7%)	117 (23.3%)	1		1	
Yes	302 (68.3%)	385(76.7%)	0.68 (0.50-0.92)	**0.02**	0.75 (0.53-1.03)	0.08
**Previous hospitalisation**						
No	267 (55.5%)	320 (59.7%)	1			
Yes	214 (44.5%)	216 (40.3%)	1.25 (0.97-1.64)	**0.10**	-	-
**Learning difficulties**						
No	402 (83.1%)	400 (74.3%)	1		1	
Yes	82 (16.9%)	138 (25.7%)	0.59 (0.43-0.81)	<0.001	**0.63 (0.44-0.89)**	**0.01**
**Neurological deficits**						
No	420 (86.8%)	462 (85.7%)	1			
Yes	64 (13.2%)	77(14.3%)	0.92 (0.65-1.30)	0.62	-	-
**Frequent seizures**						
No	416(86.1%)	480 (88.7%)	1			
Yes	67(13.9%)	61 (11.3%)	1.25 (0.86-1.84)	0.24	1.26 (0.82-1.92)	0.26
**Focal seizures**						
No	259 (53.5%)	269 (49.7%)	1			
Yes	225 (46.5%)	272 (50.3%)	0.83 (0.63-1.10)	0.16	0.84 (0.61-1.12)	0.23
**Status epilepticus**						
No	304 (68.6%)	393 (80.2%)	1		1	
Yes	139(31.4%)	97 (19.8%)	1.82 (1.33-2.53)	<0.001	**1.98 (1.40-2.88)**	**<0.001**

*****lack of previous hospitalisation was associated with lack of burn marks and was therefore not entered in the multivariable model. IQR = interquartile range; CI= confidence interval.
